# Compliance level toward COVID-19 preventive measures and associated factors among the Ambo University community, 2021

**DOI:** 10.3389/fpubh.2022.958270

**Published:** 2022-09-27

**Authors:** Ephrem Yohannes Roga, Gemechu Gelan Bekele, Dajane Negesse Gonfa

**Affiliations:** Department of Midwifery, College of Medicine and Health Sciences, Ambo University, Ambo, Ethiopia

**Keywords:** COVID-19, Ambo University, compliance, practice, attitude, knowledge

## Abstract

**Background:**

The coronavirus illness is a highly contagious viral infection with serious health consequences that has occurred all around the world. Application of COVID-19 prevention precautions and social interventions by the general public are critical to successfully combating the epidemic. Therefore, this study aimed to determine the level of compliance and associated factors with COVID-19 prevention strategies.

**Methods:**

A facility based cross-sectional study was conducted from June 01 to August 30, 2021 on a sample of 380 randomly selected Ambo University community members. A self-administered structuralized questionnaire was used to collect the data. The determining factors with the level of compliance were finally identified using a multivariate logistic regression model. The association was reported using odds ratios with a 95% CI, and significance was declared using a *P* < 0.05.

**Results:**

A total of 378 respondents participated in the study with a response rate of 98.9%. Most of the respondents, 75.7%, 57.9%, 47.4%, 61.9% had good knowledge, favorable attitude, good practice and good compliance toward COVID-19 preventive measures, respectively. In participants aged between 18 and 30 [AOR = AOR = 2.23, 95% CI: (1.13, 4.41)], good knowledge [AOR = 2.64, 95% CI: (1.46, 4.78)], favorable attitude [AOR = 4.5, 95% CI: (2.63, 7.71)], and good practice [AOR = 2.98, 95% CI: (1.82, 4.89)] were significantly associated with good compliance toward COVID-19 preventive measures.

**Conclusion and recommendation:**

Despite the fact that COVID-19 is a global and national priority, the preventive measures were not sufficiently followed. Therefore, it is essential to continue working on the community's knowledge, practices, and attitude about COVID-19 preventive measures through media campaigns, which will ultimately increase compliance. Additionally, the concerned stakeholders should consider the required interventions for the strongly associated factors that have been discovered in this current study.

## Background

Coronavirus disease 2019 (COVID-19) is a highly contagious illness that has killed many people across the globe (1). As of 7^th^ May 2022, globally, there have been 600,875,353 confirmed cases of COVID-19, including 6,486,034 deaths, and a total of 11,562,157,794 vaccine doses have been administered. In Ethiopia (out of a total population of 116.4 million) 493,167 people have been confirmed for COVID-19, with over 7,571 deaths (2).

The impact of the COVID-19 outbreak on low-income countries including Sub-Saharan Africa is expected to be far-reaching and more disastrous than in high-income countries, due to pre-existing conditions such as population size, health system status, and health workforce, which are expected to exacerbate any COVID-19-related health outcomes (3, 4). According to a study conducted in Ethiopia, the pandemic has severely impacted the academic and business operations of higher education institutions by dwindling their sources of income, lowering employee productivity, and limiting institutional capacity to cover key costs such as salary and rent (5).

The WHO has devised a number of mitigation techniques to curb the spread of COVID-19 due to its pandemic nature and lack of effective treatment. Staying at home, social distancing, wearing masks, and practicing hand hygiene are some of the most frequently advised pandemic prevention techniques. Furthermore, in reaction to the pandemic, countries all over the world have taken numerous measures to halt the virus' spread and protect vulnerable people from infection (6). These strategies are critical for lowering mortality and alleviating the burden on healthcare systems (4, 7). Such safeguards are thought to reduce COVID-19 transmissions in general and in particular to protect those at higher risk of severe illness, such as the elderly and those with underlying medical conditions like diabetes mellitus (8), and in frontline health workers (9, 10).

Moreover, in order to optimize the vaccination program and achieve a notable success in the COVID-19 immunization program across the continent, these remedies and other crucial recommendations to these problems were offered (11). A thorough and timely planning process, prompt plan implementation, rigorous community involvement, and a strong multi-sector partnership all contributed to the success of the COVID-19 vaccination campaign in Africa (12).

Even though people had high knowledge, gaps and laxity have been observed in the application of preventive measures for COVID-19 among citizens (13–15). New cases have continued to emerge despite efforts to expand public health interventions to contain and control the spread of the coronavirus throughout the world. Moreover, a study done in India revealed that on April 25, 2020, the recovery rate from COVID-19 was 21.97%, and by June 1, 2020, it was 79%. Furthermore, the authors forecast a monthly percentage increase in the number of COVID-19 cases from May 1st to December 1st, 2020. This analysis would enable the relevant authorities to implement effective preventive measures during the decision-making process (16). According to the study conducted in India, France, China, and Nepal to predict the coronavirus outbreak, the number of COVID-19 cases will increase gradually and the epidemic will continue, but the number of active cases has been drastically reduced in three of these countries with France being the exception (17).

Currently, there is no conclusive cure or specific antiviral therapeutics suggested for preventing or treating COVID-19. Thus, preventive measures ranging from individuals to large-scale societal level practices are the only available means to control the spread of the virus and minimize its impacts (18, 19). So, the aim of this study was to assess the compliance level of the Ambo University community toward prevention measures for COVID-19. The finding of this study might help the higher administrators, policy makers, researchers, and concerned stakeholders review their guidelines to contain the pandemic and take appropriate measures against those who break the prevention protocol. Therefore, this study aimed to determine the level of compliance and associated factors with regard to COVID-19 prevention measures at Ambo University, in 2021.

## Methods

### Study area and period

This study was conducted at Ambo University campuses from June 01 to August 30, 2021. Ambo University is located in Ambo, the capital of West Shaw Zone of Oromia Regional State. According to the information from the Office of Vice President for Administration and Student Services, Ambo University was established in 1947 as the School of Agriculture, the first agricultural school in Ethiopia. Ambo University is one of the foremost higher education institutions boasting significant contributions to the country‘s overall development by producing skilled human resources in various fields. Currently, the university runs 48 graduate and 70 undergraduate programs. The programs are classified into nine colleges/institutes/schools with several academic departments. Besides the main campus at Ambo, the university has three campuses at Awaro, Guder, and Woliso. The university also owns three research centers; two of them (one at Ejere and the other at Bilo) focus on research and conservation of indigenous trees, while the third at Birbirsa (about 20 km from Ambo) works on overall agricultural research and development and was named after a prominent Ethiopian humanitarian, Abebech Gobena.

### Study design

An institution-based perspective cross-sectional study was conducted among the Ambo University community.

### Study population

The study was comprised of all Ambo University employees, both academic and administrative, who provided services at Ambo University during the study period. Those who were unable to reply due to illness or who refused to engage in the study were omitted.

### Sample size determination and sampling procedure

Because no studies had been undertaken in this area, the sample size was calculated using a single population proportion formula with a confidence level of 95%, significance level of 1.96, 5% margin of error (d), and a prevalence of compliance level (*P* = 50%). The population correction formula was utilized in this study because the population (3,469) was <10,000. After adding 10% to account for the non-response rate, the final sample size was 380. Two campuses out of four were chosen at random, ensuring that at least 30% of Ambo University's campuses were covered. As a sampling frame, a list of employed attendance content comprising both administrative and academic staff was used. As a result, the participants of this study were chosen using a simple random sampling technique with a lottery method.

### Study variables

#### Independent variables

Socio-demographic factors (**s**ex, age, religion, educational level, marital status**, e**thnicity, family size, and average monthly income), practice, knowledge, and attitude were independent variables.

#### Dependent variable

Compliance level toward COVID-19 preventive measures.

### Operational definitions

#### Compliance level toward COVID-19 preventive measures

The score for compliance-related questions was 18 points and respondents who rated a sum score of 50% and above were considered to have good compliance and those who rated below 50% were considered to have poor compliance (20).

#### Knowledge toward COVID-19 preventive measures

There were 22 knowledge-related questions and respondents with a sum score of 50% or more were regarded to have strong knowledge, while those with a score of <50% were considered to have poor knowledge (21).

#### Attitude toward COVID-19 preventive measures

The score for attitude-related questions was 16 points and respondents who rated a sum score of 50% and above were considered to have a favorable attitude and those who rated below 50% were considered to have an unfavorable attitude (22).

#### Practices toward COVID-19 preventive measures

Practices-related questions were scored out of 14 possible points, with respondents with an overall score of 50% or higher being considered to have good practices, and respondents with an overall score of <50% were considered to have poor practices (23).

### Data collection technique and tool

The data were gathered by a self-administered questionnaire devised by the investigators after reading several COVID-19 guidelines and other sources (24–26). The questionnaire was divided into four sections. The study contained questions about respondents' socio-demographic factors, attitudes, practices, knowledge, and compliance toward COVID-19 preventive measures. The data collection tool was originally written in English, then translated into Afaan Oromo (the local language), and finally back to English by language experts to ensure its consistency. In addition, four trained BSc nurses (two data collectors for each campus) and three MSc-holder nurses oversaw the data collection process.

### Data quality assurance

This study recruited experienced data collectors to assure data quality. The questionnaire was developed by the lead investigator using questions from previously published peer-reviewed studies. The pretest was conducted on 5% of the sample size at Guder Campus. For one day, data collectors and supervisors received training on research objectives, data collection tools and processes, and interview techniques. Furthermore, each supervisor was responsible for overseeing the data collection process on a daily basis.

### Data processing and analysis

The data were double-checked for accuracy and completeness before being entered into Epinfo version 7.2.2.6. The data were then cleaned, coded, and analyzed using SPSS version 25. Missed values and outliers were checked in the data. To define the required variable, descriptive analysis (such as frequencies, tables, percentages, means, and standard deviation) was used. Then, based on bivariate logistic analysis, any variables with a *P* < 0.25 were considered candidates for multivariate logistic regression models. The factors associated with compliance level to COVID-19 preventive measures were identified using multivariable logistic regression at the 95% confidence level. A significance level of 0.05 was taken as a cutoff value for all statistical significance tests. Multi-collinearity was checked between each variable using the variance inflation factor. Accordingly, no multi-collinearity was detected. Hosmer and Lemeshow's goodness of fit test was administered to check the model fitness.

## Results

### Socio-demographic characteristics of the participants

In this study 378 participants completed the questionnaire, translating to a 98.95% response rate. The sample consisted of 51.3% (*n* = 194) males, with a median age of 29.9 years (SD ± 4.98), with the majority aged (43.1%, *n* = 140) between 31 and 40 years. Participants with bachelor's degrees constituted 40.7% (*n* = 154) and the highest educational achievement in the sample was a PhD degree. More than half (55.8%, *n* = 211) of the respondents were from the Oromo ethnic group. The majority of the participants, 50.8% (192), were followers of a protestant religion. Moreover, most of the respondents', 56.9% (*n* = 215), average monthly income was between 4000 and 9000 Ethiopian Birr. In terms of the respondents' marital status, 148 (39.2%) of them were married. The majority of the participants', 215 (56.7%), had a family size of 1–4 in number (Table 1).

**Table 1:** Socio-demographic characteristics of the Ambo University community participating in the study, Ethiopia, 2022.

**Variables**	**Category**	**Frequency**	**Percent**
Sex	Male	194	51.3
	Female	184	48.6
Age	18–30	140	37.0
	31–40	163	43.1
	≥41	75	19.8
Ethnicity	Oromo	211	55.8
	Amhara	152	40.2
	Tigre	10	2.6
	Gurage	5	1.3
Religious	Orthodox	165	43.7
	Protestant	192	50.8
	Muslim	10	2.6
	Catholic	11	2.9
Averagely monthly income	1,200–3,000	89	23.5
	4,000–9,000	215	56.9
	10,000–15,000	74	19.6
Educational level	Diploma	86	22.8
	First degree	154	40.7
	Second degree	104	27.5
	PhD/Assistant professor	34	9.0
Marital status	Single	117	31
	Married	148	39.2
	Divorced	76	20.1
	Widow	37	9.8
Family size	1–4	215	56.9
	5–8	126	33.3
	9–15	37	9.8

### Knowledge, attitude, practice, and compliance of respondents toward COVID-19 preventive measures

The composite of overall knowledge of the participants showed that a majority of the respondents, 286 (75.7%), have good knowledge about COVID-19 preventive measures. Additionally, more than half, 219 (57.9%), of the participants show a favorable attitude toward COVID-19 preventive measures. Moreover, the current study shows that less than half of the participants, 179 (47.4%), have good practices regarding COVID-19 preventive measures. Additionally, a majority, 234 (61.9%), of the participants have good compliance toward COVID-19 preventive measures. Most of the study participants, 196 (51.9%), report that they wear a facemask frequently. Most of the respondents, 240 (63.5%), did not follow advice to avoid consuming outdoor food in order to prevent contracting and spreading COVID-19 (Table 2).

**Table 2:** Preventive practices of COVID-19 among the Ambo University community, 2022 (*n* = 378).

**Preventive practices of COVID-19 characteristics**	**Category**	**Frequency**	**Percent**
In order to prevent contracting and spreading COVID-19, I avoid going out of my home	No	183	48.4
	Yes	195	51.6
In order to prevent contracting and spreading COVID-19, I avoid unnecessary vacations	No	179	47.4
	Yes	199	52.6
In order to prevent contracting and spreading COVID-19, I avoid consuming outdoor food	No	240	63.5
	Yes	138	36.5
In order to prevent contracting and spreading COVID-19 I avoid handshaking, hugging, and kissing	No	187	49.5
	Yes	191	50.5
In order to prevent contracting and spreading COVID-19, I avoid public transportations (taxi, bus, plane, train)	No	203	53.7
	Yes	175	46.3
In order to prevent contracting and spreading COVID-19, I avoid going to work	No	191	50.5
	Yes	187	49.5
In order to prevent contracting and spreading COVID-19, I frequently wash my hands	No	211	55.8
	Yes	167	44.2
In order to prevent contracting and spreading COVID-19, I pay more attention to my personal hygiene than usual	No	192	50.8
	Yes	186	49.2
In order to prevent contracting and spreading COVID-19, I use disinfectant and solutions	No	194	51.3
	Yes	184	48.7
In order to prevent contracting COVID-19, I use herbal products and traditional medicine	No	218	57.7
	Yes	160	42.3
In order to prevent contracting COVID-19, I take vitamin supplements	No	218	57.7
	Yes	160	42.3
In order to prevent contracting and spreading COVID-19, I use facial masks frequently	No	182	48.1
	Yes	196	51.9

### Factors associated with compliance toward COVID-19 preventive measures among the Ambo University community

Bivariate logistic analysis identified candidate variables such as age, educational status, sex, family size, knowledge, attitude, and practices which were associated with compliance of COVID-19 preventive measures at *P* < 0.25. Then multivariate logistic analysis was conducted and found to be a good fit for these factors. At a *P* < 0.05, multivariate logistic regression analysis revealed that age, knowledge, attitude, and practice were considerably and statistically associated with compliance to COVID-19 preventive measures. Younger respondents were two times more likely to have good compliance toward COVID-19 preventive measures than those >40 years old. Participants with good knowledge, favorable attitude, and good practice were 2.6, 4.5, and 2.9 times respectively more likely to have good compliance toward COVID-19 preventative measures than their counterparts (Table 3).

**Table 3:** Bivariate and multivariate logistic regression of factors affecting respondents' compliance toward COVID-19 preventive measure among the Ambo University community, 2022, (*n* = 378).

**Variable**		**Compliance**				**COR (95% CI)**	**AOR (95%CI)**	***P*-value**
		**Good**		**Poor**				
		**Nu**	**%**	**Nu**	**%**			
Age	18–30	91	65	49	35	1.71 (0.97, 3.03)	**2.23 (1.13, 4.41)**	**0.001**
	31–40	104	63.8	59	36.2	1.63 (0.94, 2.83)	1.67 (0.88, 3.16)	
	≥41	39	52	36	48	1.00	1.00	
Educational status	Diploma	39	45.3	47	54.7	1.00	1.00	
	First degree	101	65.6	53	34.4	2.29 (1.34, 3.94)	1.59 (0.84, 3.01)	
	Second degree	67	64.4	37	35.6	2.18 (1.22, 3.91)	0.79 (0.37, 1.68)	
	PhD (Assistant Professor)	27	79.4	7	20.6	4.65 (1.83, 11.8)	1.35 (0.44, 4.21)	
Knowledge	Good	194	67.8	92	32.2	2.74 (1.69, 4.45)	**2.64 (1.46, 4.78)**	**0.003**
	Poor	40	43.5	52	56.5	1.00	**1.00**	
Attitude	Favorable	168	76.7	51	23.3	4.64 (2.98, 7.24)	**4.5 (2.63, 7.71)**	**0.004**
	Unfavorable	66	41.5	93	58.5	1.00	1.00	
Practice	Good	137	76.5	42	23.5	3.43 (2.2, 5.35)	**2.9 (1.82, 4.89)**	**0.001**
	Poor	97	48.7	102	51.3	1.00	1.00	

Bold values indicate that the variables are significantly associated with the compliance towards COVID-19 preventive measures.

## Discussion

This study found that most of the respondents, 61.9% (95% CI: 56.9, 66.7), had good compliance toward COVID-19 preventive measures. Moreover, the current study revealed that age, knowledge, attitude, and practice were identified as important factors associated with compliance toward COVID-19 preventive measures in the study area. This finding is in line with the study conducted in Ethiopia that found that about 55.3 and 57.8% had good compliance toward COVID-19 preventive measures (21). Another study conducted in Uganda showed that 74% had good compliance toward COVID-19 preventive measures (27). A study conducted in Debre Birhan, Ethiopia showed that 56.1% of women exhibited good compliance with COVID-19 preventive measures (28). A study conducted in South Korea also showed that participants' overall compliance rate was 50.5% (29). However, the study conducted in Southeastern Ethiopia showed the overall good compliance and knowledge of health professionals regarding COVID-19 preventive measures were 21.6 and 25.5%, respectively (30). Despite the current finding, a comparative cross-sectional study between the developed and developing countries demonstrated that the overall compliance to COVID-19-related preventive measures was poor (31). A study done in Gondar indicated that nearly half of the study participants [48.96% (95% CI: 45.05%, 52.89%)] had poor adherence toward COVID-19 mitigation measures (23). This could be related to differences in study design, socioeconomic status, and sample size. In comparison to prior research, the current data demonstrated that compliance with COVID-19 prevention strategies was enhanced. This could be because information regarding COVID-19 prevention methods has been widely disseminated through various media. The current study, however, is not entirely satisfied with the findings, indicating that there is still a gap that needs to be filled.

The current study showed that younger people are more compliant than older ones. This finding is supported with the evidence from a study conducted in Ethiopia (28). Adults and people in their later years have the lowest compliance rates in both developing and wealthy countries, according to a study. Older adults are often reliant on other family members and may not be up to date on current events, which reduces their compliance (31). This could be because, at this age, people's conduct is still largely governed by extrinsic motivation (reward–punishment) and is focused on achieving quick short-term goals (32) rather than social awareness. Furthermore, people who view regulations as being too stringent or demanding are more likely to break them (33), which may be particularly common in this age range. However, according to a study conducted in Spain, the younger group was less compliant than the older group (34). A study conducted in Ghana found that strong COVID-19 prevention strategies were favorably linked with older women (35). According to studies, advanced maternal age is a risk factor for severe complications and mortality connected to COVID-19 preventative measures during pregnancy, which explains why this group of people adheres to them well (36). According to a study conducted in Ethiopia, younger people were more knowledgeable than elderly people (20). As a result, there may be a difference due to the level of knowledge, understanding, and recalling evidence about COVID-19 preventive actions being better among younger people than among older people. This could lead to younger age groups having a better awareness of COVID-19 preventive actions and the severity and implications of contracting the illness, resulting in better compliance with preventive measures.

The current study found that compliance toward COVID-19 preventive measures was highly linked to participants' positive attitudes. This conclusion is backed up by a cross-sectional study conducted in 12 Asian nations, which found that good sentiments toward COVID-19 prevention measures were linked to high compliance (37). The study conducted in Gondar, Ethiopia showed that the respondents who had a favorable attitude toward COVID-19 preventive measures were 2.54 times more likely to adhere to the mitigation measures than respondents who had an unfavorable attitude toward COVID-19 preventive measures (23). This is corroborated by the findings of a qualitative method study, which found that social variables, such as negative attitudes toward persons who practice prevention measures, were the main reasons for people not following the prevention measures. People with a negative attitude regard a person who wears a facemask and uses hand sanitizer as aliens or as those who are afraid of death. As a result, people do not take preventative precautions in order to avoid being classified as such. Furthermore, a key source from the Borena Health Office stated that “stigma is one of the elements that has influenced the usage of preventative methods. “Furthermore, some believe that the country is free of coronavirus” (20). One possible explanation is that those who have a positive attitude about COVID-19 prevention measures trust the science of mitigation measures and follow the guidelines' directions.

This study discovered that having a solid understanding of COVID-19 preventive measures is highly linked to good compliance with COVID-19 preventive measures. Participants with a low degree of understanding of COVID-19 preventive measures had a decreased chance of adhering to COVID-19 preventive measures. A study conducted elsewhere supports knowledge as the product of awareness based on receiving relevant information (20). According to a study conducted in Southeast Ethiopia, participants with adequate awareness of COVID-19 prevention strategies had a roughly 1.8 times better likelihood of good compliance. This could be due to proper instruction (training duration), the availability of reading materials/internet connections, and personal commitments (30). Furthermore, another study conducted in China found a link between knowledge of COVID-19 preventive measures and compliance with COVID-19 preventive measures (38). Moreover, a study conducted in Nigeria found that a lack of awareness can have a negative impact on the level of compliance with coronavirus prevention methods (39).

The current study found that the participants' level of compliance with COVID-19 prevention measures is influenced by their practice. Participants who have a high degree of practice also have a high level of compliance with the COVID-19 preventive measure. This conclusion is consistent with a research study conducted in Nigeria (40). Individual and governmental preventive measures' perceived effectiveness had a significant impact on PPM compliance. Other research studies have emphasized the importance of believing that preventive actions will be effective (41). As a result, the advertisements were successful in boosting public knowledge about the effectiveness of preventive measures in reducing COVID-19. Subsequently, greater resources for such efforts should be allocated (42). Furthermore, the other study found that good preventive practices were substantially linked to high COVID-19 preventative measure compliance (37).

This current study might be exposed to a social desirability bias due to the nature of the study. As a result of this limitation, the findings of this study should be interpreted with caution. However, this weakness was attempted to be minimized by observing participants' observable compliance dimensions immediately following the interview and correcting responses as needed. Furthermore, the lack of prior studies on compliance with COVID-19 prevention and control strategies in various settings restricted the discussion of this study to the available circular guidelines. This may have jeopardized the findings' comparability and generalizability, and readers should be aware of this limitation.

## Conclusion and recommendation

The overall level of compliance with COVID-19 prevention measures has been assessed to be satisfactory. Age, knowledge, attitude, and practice have all been associated to a high level of compliance with COVID-19 prevention measures. Furthermore, all stakeholders should consider the interventions that are required for the identified highly connected factors. These findings point to the need for programs and policies to increase people's understanding, attitudes, and COVID-19 preventive activities in Ethiopia. Furthermore, the concerned entities should adopt actions to enhance adherence to COVID-19 preventive measures.

## Language plain summary

Coronavirus disease is a highly contagious viral infection with major health consequences that has been introduced globally. The general public's compliance with public health and social initiatives is important to successfully combatting the epidemic. The goal of this study was to investigate the level of COVID-19 prevention strategy compliance and associated factors. A facility-based cross-sectional study of 380 randomly selected Ambo University community members was undertaken from June 1 to August 30, 2021. To collect data, a self-administered structured questionnaire was used. Using a multivariate logistic regression model, the determining factors affecting the level of compliance were finally discovered. According to the findings, the majority of respondents had good knowledge, a positive attitude, good practice, and good compliance with COVID-19 prevention measures. Participants aged 18–30, who had a positive attitude and good practice, were associated with good compliance with COVID-19 prevention strategies. The overall level of COVID-19 prevention knowledge, attitude, and compliance was deemed to be satisfactory. Age, knowledge, attitude, and practice were all found to be substantially linked to successful COVID-19 prevention compliance. As a result, all stakeholders should evaluate the necessary interventions for the identified highly-linked factors.

## Data availability statement

The original contributions presented in the study are included in the article/supplementary material, further inquiries can be directed to the corresponding author/s.

## Ethics statement

The studies involving human participants were reviewed and approved by Ambo University Institutional Review Board. The patients/participants provided their written informed consent to participate in this study.

## Author contributions

ER and GB contributed significantly to the conceptualization, design of the project, and contributed to the article's development or critical revision for essential intellectual content. DG assisted with data collecting, handled data analysis, and interpretation. All authors agreed to be responsible for all elements of the work, agreed to submit to the current journal, and gave final approval of the published version.

## Conflict of interest

The authors declare that the research was conducted in the absence of any commercial or financial relationships that could be construed as a potential conflict of interest.

## Publisher's note

All claims expressed in this article are solely those of the authors and do not necessarily represent those of their affiliated organizations, or those of the publisher, the editors and the reviewers. Any product that may be evaluated in this article, or claim that may be made by its manufacturer, is not guaranteed or endorsed by the publisher.

## Abbreviations

COVID-19: Coronavirus disease 2019
HCWs: Health Care Workers
KAP: Knowledge Attitude Practice
PPE: Personal protective equipment.
